# Synergy of two low-affinity NLSs determines the high avidity of influenza A virus nucleoprotein NP for human importin α isoforms

**DOI:** 10.1038/s41598-017-11018-1

**Published:** 2017-09-12

**Authors:** Wei Wu, Rajeshwer S. Sankhala, Tyler J. Florio, Lixin Zhou, Nhan L. T. Nguyen, Ravi K. Lokareddy, Gino Cingolani, Nelly Panté

**Affiliations:** 1 University of British Columbia, Department of Zoology, Vancouver, British Columbia V6T1Z4 Canada; 2 Thomas Jefferson University, Department of Biochemistry and Molecular Biology, Philadelphia, PA 19107 USA; 3 Institute of Biomembranes and Bioenergetics, National Research Council, Via Amendola 165/A, 70126 Bari, Italy

## Abstract

The influenza A virus nucleoprotein (NP) is an essential multifunctional protein that encapsidates the viral genome and functions as an adapter between the virus and the host cell machinery. NPs from all strains of influenza A viruses contain two nuclear localization signals (NLSs): a well-studied monopartite NLS1 and a less-characterized NLS2, thought to be bipartite. Through site-directed mutagenesis and functional analysis, we found that NLS2 is also monopartite and is indispensable for viral infection. Atomic structures of importin α bound to two variants of NLS2 revealed NLS2 primarily binds the major-NLS binding site of importin α, unlike NLS1 that associates with the minor NLS-pocket. Though peptides corresponding to NLS1 and NLS2 bind weakly to importin α, the two NLSs synergize in the context of the full length NP to confer high avidity for importin α7, explaining why the virus efficiently replicates in the respiratory tract that exhibits high levels of this isoform. This study, the first to functionally characterize NLS2, demonstrates NLS2 plays an important and unexpected role in influenza A virus infection. We propose NLS1 and NLS2 form a bipartite NLS *in trans*, which ensures high avidity for importin α7 while preventing non-specific binding to viral RNA.

## Introduction

Influenza A virus is a major worldwide threat to human health with limited antiviral therapeutics in place. Currently, only two classes of influenza antiviral drugs are available, which target viral surface proteins. Unlike surface proteins, which are highly variable between strains and mutate easily, the nucleoprotein (NP) of influenza A virus is a highly conserved protein with roles in several stages of the influenza infectious cycle^1^. Thus, an antiviral targeting NP is likely to be effective against a wide variety of different influenza viruses. The primary function of NP is to encapsidate the viral genome, consisting of eight segments of single-stranded negative-sense RNA, into viral ribonucleoprotein complexes (vRNPs)^1^. The RNA segments range in length from 890 to 2,341 nucleotides and contain approximately one NP per 24 nucleotides of RNA or between 3 to 8 dozen copies of NP per RNA segment^2, 3^. During the early stages of influenza infection, vRNPs are released in the cytoplasm and imported into the nucleus of infected cells for viral replication^4^. The nuclear import of vRNPs is mediated by NP^4, 5^. After initial vRNA transcription, NP is synthesized in in the cytoplasm of infected cells as early as 2 h after infection^6^ and then enters the nucleus to help in the replication of full-length viral genome segments^7^ and to assemble progeny vRNPs^4^.

**Figure 1:**
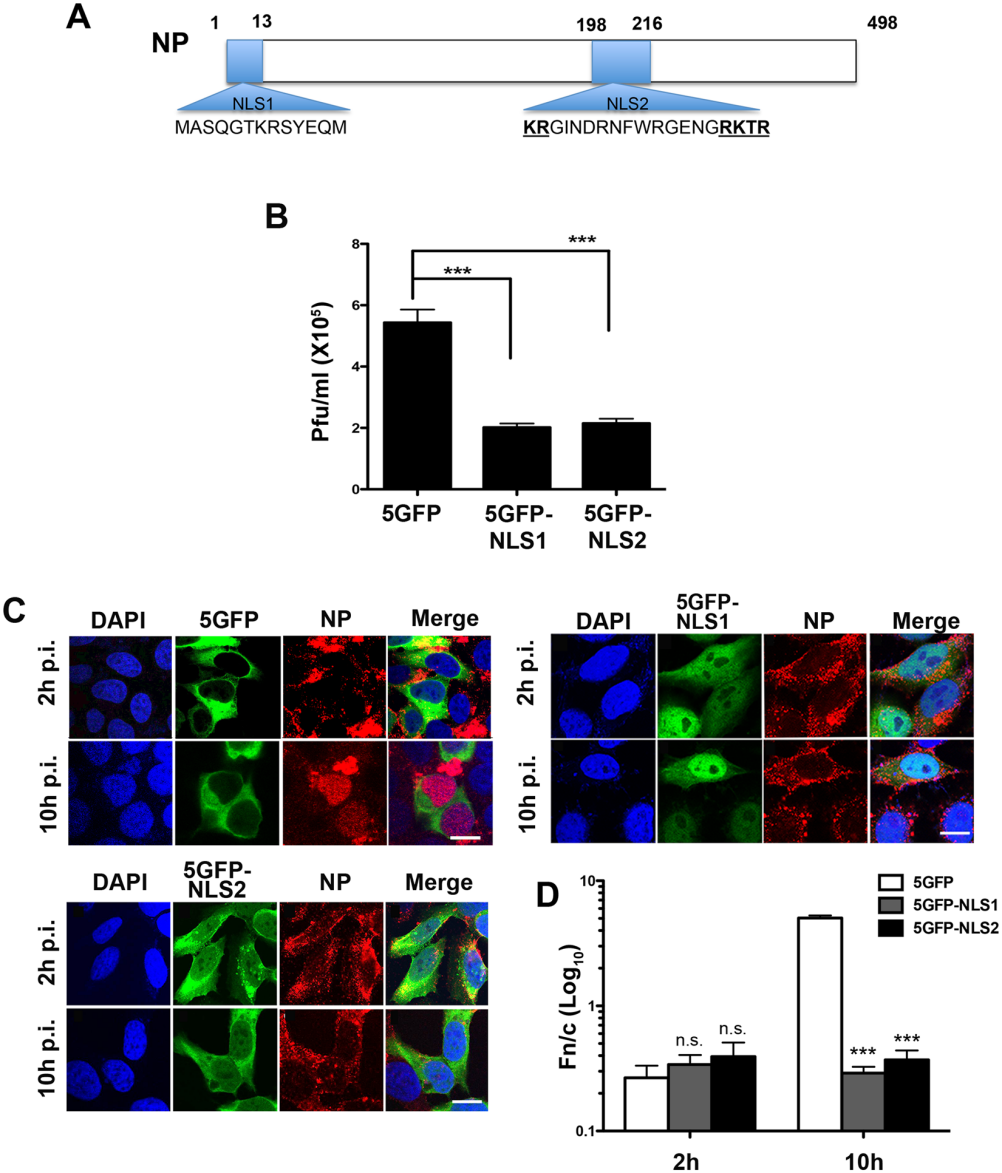
NP NLS2 contributes to nuclear import during influenza A virus infection. (**A**) Schematic diagram of the location of NLS1 and NLS2 on influenza A NP. The amino acid sequences is based on influenza A virus, strain X-31, A/Aichi/68 (H3N2). Numbers indicate amino acids positions. (**B**) HeLa cells expressing 5GFP, 5GFP-NLS1, or 5GFP-NLS2 were infected with influenza A virus (X-31). At 24 h post-infection, the supernatant was collected and the viral titer was determined by infecting MDCK cells and counting plaques 3 days post-infection. Bar graphs show the mean ± standard error of the mean from three independent experiments (***p < 0.001, one-way ANOVA followed by Tukey’s tests). (**C**) Competition of nuclear import of NP by NLS1 and NLS2 during an infection. HeLa cells expressing 5GFP, 5GFP-NLS1, or 5GFP-NLS2 were infected with influenza A virus for 2 or 10 h and the subcellular localization of 5GFP and NP were analyzed by confocal fluorescence microscope after immunolabeling NP (red) using a specific antibody. DAPI (blue) was used to observe the nucleus. Scale bars, 10 µm. (**D**) Quantification of the ratio of nuclear to cytoplasmic fluorescence (Fn/c) of NP, corrected for background fluorescence, for experiments shown in **C**. Shown are the means ± standard error of the means scored from three independent experiments. (***p < 0.001, one-way ANOVA followed by Tukey’s tests). The levels of expression of 5GFP, 5GFP-NLS1, and 5GFP-NLS2 for experiments in Fig. 1B and C are shown in Fig. S2.

Nuclear import is a highly selective process that requires a nuclear localization sequence (NLS) on the import cargo^8, 9^. NLSs are recognized by soluble nuclear transport receptors of the importin β/karyopherin β superfamily^10^. The best-characterized NLSs, often referred to as “classical” NLSs, contain either monopartite or bipartite stretches of basic amino acids that are recognized by the adapter importin α, which binds the receptor importin β/karyopherin β1 to transport the trimeric NLS-cargo:importin α/β complex into the nucleus^9, 11^. NPs from all strains of influenza A viruses contain two NLSs (Fig. 1A): NLS1 at the N-terminus^12, 13^ and NLS2 spanning residues 198–216^14^. A third overlapping bipartite NLS, at amino acids 90–121 of NP, has been found in only nine influenza A strains^15^. NLS1 is an unconventional NLS^12^ that binds weakly to the minor NLS-binding pocket of importin α^16^ and is inactivated by phosphorylation to prevent re-import of viral RNPs at the late stage of infection^17, 18^. To date, several studies have characterized NLS1^13, 16, 19–21^, whereas NLS2 is less well understood and the few reports about this second nuclear import signal are contradictory.

NLS2 was first discovered through sequence alignment of the Thogoto virus NP with influenza A virus NP^14^. It contains two basic amino acid clusters separated by a 13 amino acid linker region (Fig. 1A) and, therefore, it was hypothesized to be a putative bipartite NLS^14^. However, the crystal structure of trimeric NP suggested these two basic clusters are only 15 Å apart^22^, too close to bind to importin α as a stretched bipartite polypeptide chain, thus arguing against the idea that NLS2 is a genuine bipartite import signal^22^. A mutational study of NLS2 suggested this import signal is not essential for nuclear import of the influenza genome^21^. However, through antibody inhibition and peptide competition experiments, others found that NLS2 could mediate the nuclear import of vRNPs^23^. Therefore, more studies are needed to elucidate the effective role of NLS2 in the nuclear import of the viral genome during influenza infection.

In this study, we investigate the role of NLS2 of NP in the nuclear import of influenza A virus. We also evaluate whether NLS2 is a classical bipartite NLS through mutational and crystallographic analysis of two naturally occurring variants of NLS2. Our results demonstrate that NLS2 is an atypical monopartite NLS that binds weakly to the major NLS-binding site of importin α and synergize with NLS1 to confer high avidity for certain importin α isoforms.

## Results

### NLS2 contributes to nuclear import during influenza A infection

To evaluate the contribution of NLS2 during influenza A virus infection, cells expressing a chimeric protein containing NLS2 fused to the C-terminus of five GFP molecules in tandem (5GFP-NLS2) were infected with influenza A virus (strain A/X-31 H3N2). As a negative and positive control, cells expressing 5GFP or 5GFP-NLS1 were used, respectively. 5GFP was used because oligomers with four or less tandem copies of GFP freely diffuse into the nucleus, while 5GFP is excluded^24^. Viral production was reduced in cells expressing 5GFP-NLS2 to the same extent as in cells expressing 5GFP-NLS1 (Fig. 1B). Similar results were obtained using another influenza A virus strain (Fig. S1).

To determine whether the reduction in viral production in cells expressing 5GFP-NLS2 was due to competition of 5GFP-NLS2 with NP nuclear import during infection, cells expressing 5GFP-NLS2, 5GFP-NLS1, or 5GFP were infected with influenza A virus and NP was immunostained and localized by confocal microscopy at 2 and 10 h post-infection (p.i.). At 2 h p.i. there was no significant difference in the cellular localization of NP for all conditions (Fig. 1C,D). However, at 10 h p.i. NP was predominantly localized in the cytoplasm of NLS1- and NLS2-expressing cells, but in the nucleus of control cells expressing 5GFP (Fig. 1C,D). This suggests that both 5GFP-NLS1 and 5GFP-NLS2 compete with vRNPs/NP for nuclear import and, thus, NLS2 contributes to promote nuclear import of vRNPs/NP during influenza A infection. Although nuclear import of NP was defective in cells expressing NLS1 or NLS2, these cells had no defects on the nuclear import of a protein containing the classical SV40 NLS (Fig. S3).

### The C-terminal cluster is the main contributor to the function of NLS2

**Figure 2:**
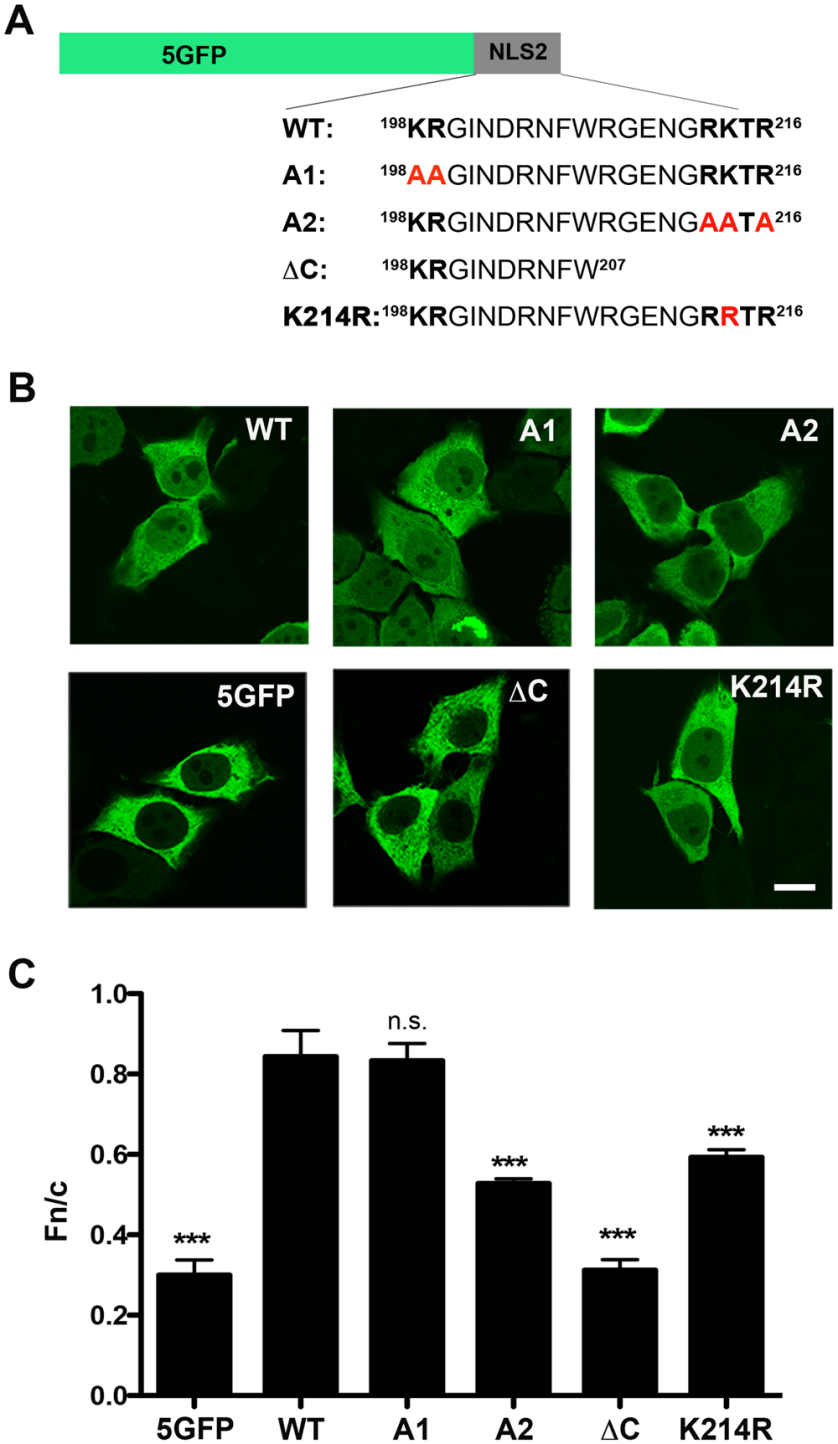
Mutational analysis of NLS2. (**A**) Schematic representation of the wild type 5GFP-NLS2 and its mutants. The two clusters of basic amino acids of NLS2 are shown in bold. Mutant A1 and A2 have alanine substitutions (shown in red bold) at the N- and C-terminal cluster, respectively. ΔC is a deletion mutant of the C-terminal of NLS2. In mutant K214R the lysine at position 214 of NP was substituted by arginine. (**B**) Confocal images of HeLa cells expressing 5GFP, wild type (WT) 5GFP-NLS2, and the mutants indicated in panel A 24 hours post transfection. Scale bar, 10 µm. (**C**) Quantification of Fn/c from the experimental conditions shown in panel B. For all panels the bar diagrams show the ratio of nuclear to cytoplasmic fluorescence (Fn/c) of NP, corrected for background fluorescence, for individual cells. Shown are the means ± standard error of the means scored from at least 85 cells for each condition from three independent experiments. (***p < 0.001, one-way ANOVA followed by Tukey’s tests).

Because NLS2 has two clusters of basic residues, it was proposed to be a classical bipartite NLS^14^. A functional bipartite NLS contains two interdependent clusters of basic amino acids both of which are essential for nuclear import^25^. To address the contribution of the two clusters of basic residues, two 5GFP-NLS2 constructs were created in which the basic residues were substituted with alanine (A): A1 mutant with two A substitutions at the N-terminal cluster and A2 mutant with three A substitutions at the C-terminal cluster (Fig. 2A). Cells transfected with the mutant plasmids revealed that the chimeric protein bearing mutations in the N-terminal cluster of basic amino acids (A1 mutant) showed similar nuclear localization as wild-type (WT) 5GFP-NLS2, while the chimeric protein bearing mutations in the C-terminal cluster of basic amino acids (A2 mutant) showed significant decrease in nuclear accumulation compared to WT NLS2 (Fig. 2B,C). Thus, the C-terminal, but not the N-terminal cluster of basic amino acids contributes to the function of NLS2, indicating that NLS2 is not a classical bipartite NLS, as previously hypothesized^14^.

Since mutations at the C-terminal basic cluster of NLS2 (A2 mutant) significantly decreased the nuclear accumulation of the chimeric protein (Fig. 2B,C), a chimeric protein with a C-terminal deletion mutant (Fig. 2A, ΔC) was generated and localized post-transfection (Fig. 2B,C). Nuclear accumulation of the ΔC chimeric protein was significantly decreased compared to that of the WT 5GFP-NLS2 with nuclear to cytoplasmic fluorescence (Fn/c) ratio similar to that of control cells expressing 5GFP (Fig. 2C). This confirms that the NLS activity of NLS2 is confined to the C-terminal cluster of basic amino acids (213-RRTR-216).

NLS2 is highly conserved between different strains of influenza A, with the noticeable difference that avian and highly pathogenic influenza A strains have an arginine at residue 214 of NP (NLS2-R) rather than a lysine (NLS2-K), as in seasonal strains. To evaluate whether this single basic amino acid difference influences nuclear import, a 5GFP-NLS2 construct with K214→ R mutation (Fig. 2A, K214R) was generated. Cells transfected with the K214R plasmid yielded lesser nuclear localization of the 5GFP chimeric protein than the wild type 5GFP-NLS2-K (Fig. 2B,C), suggesting NLS2 is more potent when position 214 is occupied by a lysine.

### NLS2 is a monopartite import signal that predominantly binds the major NLS-pocket of importin α

**Figure 3:**
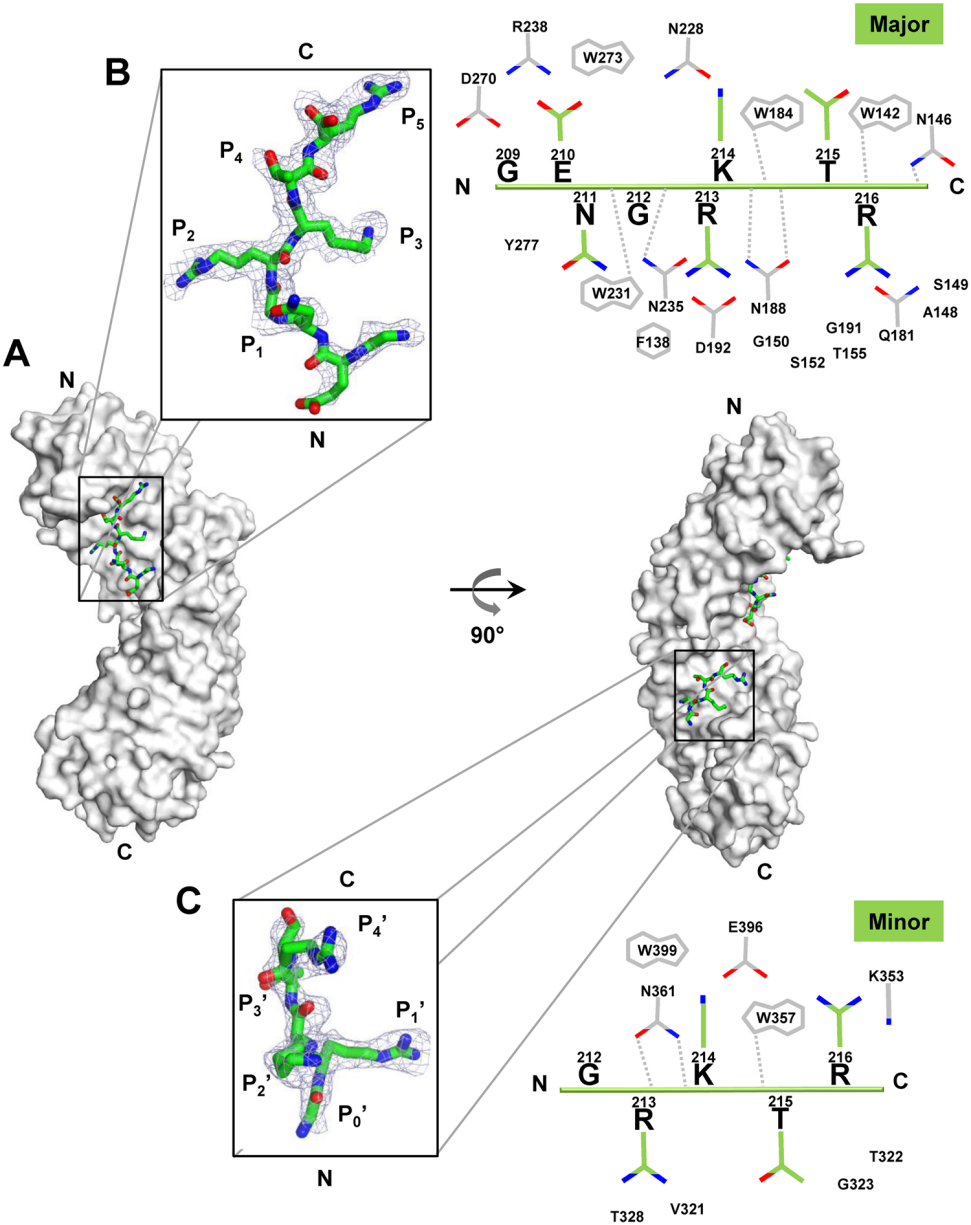
Crystal structure of ∆IBB-importin α1 bound to NP-NLS2-K. (**A**) Structure of ∆IBB-importin α1 (gray surface) in complex with NLS2-K (green sticks). (**B**,**C**) *Left panel:* zoom-in window showing an 2Fo – Fc electron density map (displayed as a blue mesh contoured at 1.25σ above background) calculated using all reflections between 15–2.25 Å resolution after omitting the NLS bound at the major (**B**) and minor (**C**) NLS-binding sites of importin α. In both cases, the density is overlaid to the NLS final model (the illustration was generated using PyMol^58^). *Right panel*: schematic diagram of the interactions NLS2-K (green) makes at the major (**B**) and minor (**C**) NLS-binding sites of importin α1 (white). Side chain nitrogen and oxygen atoms are color-colored in blue and red, respectively. Interactions between importin α side chains and the NLS main chain atoms are shown by dashed gray lines.

**Figure 4:**
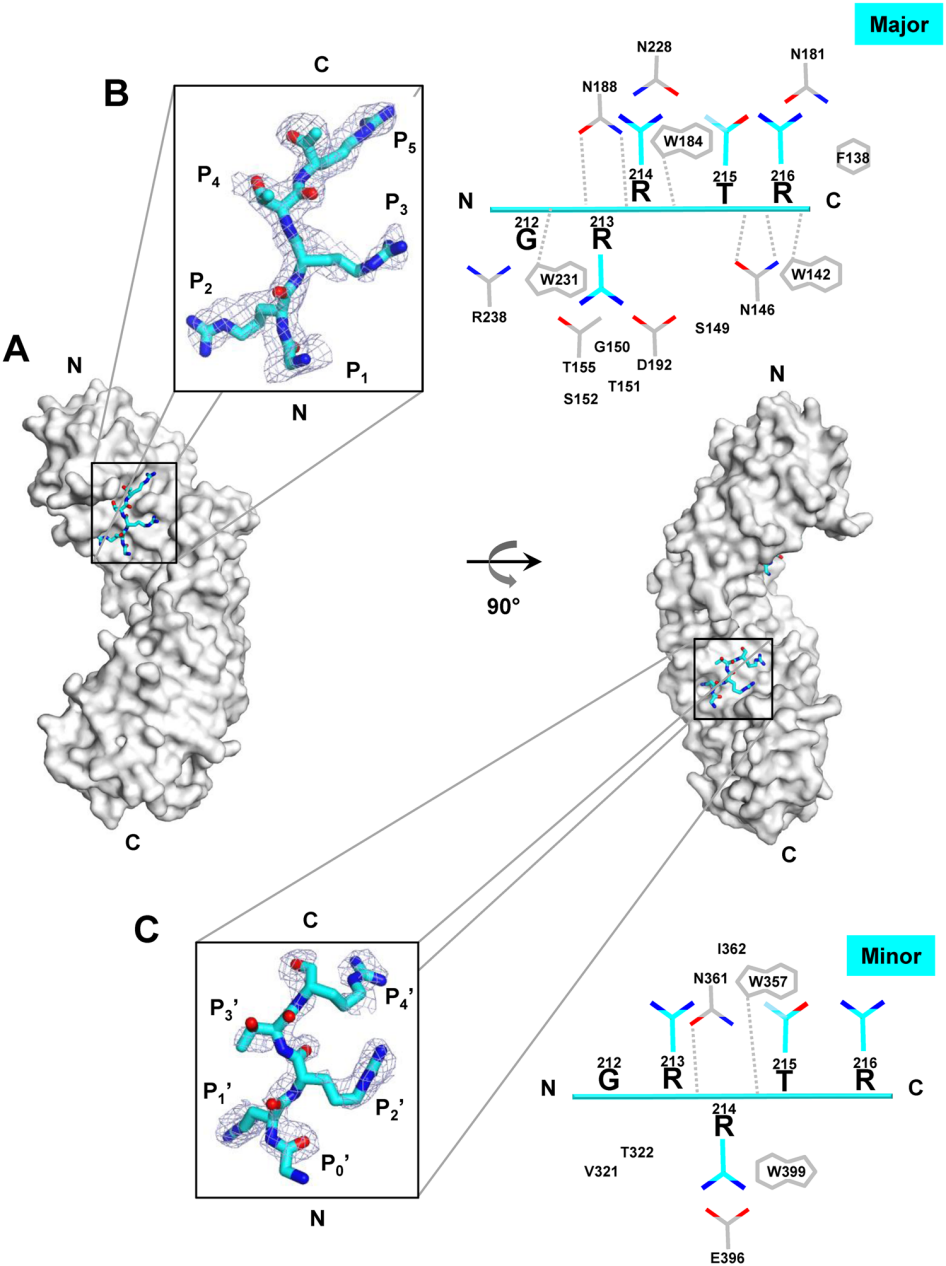
Crystal structure of ∆IBB-importin α1 bound to NP-NLS2-R. (**A**) Structure of ∆IBB-importin α1 (gray surface) in complex with NLS2-R (cyan sticks). (**B**,**C**) *Left panel:* zoom-in window showing the final 2Fo – Fc electron density map (displayed as a blue mesh contoured at 1.25σ above background) calculated using all reflections between 15–2.15 Å resolution after omitting the NLS bound at the major (**B**) and minor (**C**) NLS-binding sites of importin α. In both cases, the density is overlaid to the NLS final model (the illustration was generated using PyMol^58^). *Right panel*: schematic diagram of the interactions NLS2-R (cyan) makes at the major (**B**) and minor (**C**) NLS-binding sites of importin α1 (white). Side chain nitrogen and oxygen atoms are color-colored in blue and red, respectively. Interactions between importin α side chains and the NLS main chain atoms are shown by dashed gray lines.

To visualize the interaction of NLS2 with importin α, we co-crystallized mouse importin α1 lacking the N-terminal importin-β-binding domain^26^ (ΔIBB-importin α1) with peptides spanning NLS2-K (198-**KR**GINDRNFWRGENG**RKTR**−216) and NLS2-R (198-**KR**GINDRNFWRGENG**RRTR**-216). The crystal structures of ΔIBB-importin α1:NLS2-K and α1:NLS2-R complexes were solved by molecular replacement and refined to an Rwork/free of 17.4/20.6% and 18.0/21.0%, at 2.25 and 2.10 Å resolution, respectively (Table 1). In both structures, importin α adopts the well-known α-solenoid architecture made up of 10 stacked Armadillo (Arm) repeats, each formed by three α-helices (known as A, B and C)^27^. The Arm-core generates a continuous α-helical surface that harbors a major (Arm repeats 2–4) and a minor (Arm repeats 7–8) NLS-binding pocket, each with five points of contact for the NLS side chains, referred to as P_1_-P_5_ and P_1′_-P_5′_, respectively^27, 28^. Clear electron density for NLS2-K/R was observed at the major NLS site of importin α (Figs 3A,B and 4A,B), and, to a lesser extent, at the minor NLS-pocket (Figs 3C and 4C). We unambiguously interpreted 8 residues for NLS2-K (Fig. 3A,B) at the major NLS-pocket and only 5 for NLS2-R (Fig. 4A,B), supporting the idea that a K at position 214 of NP-NLS2 results in higher affinity for importin α. The residues visible at the major site correspond to the C-terminal basic box of NLS2, which validates the mutagenesis analysis in Fig. 2B,C, and confirms NLS2 is a monopartite NLS. Likewise, in both complexes only five residues of NLS2, the same that bound at the major site, were visible at the minor NLS-binding site (212-GRK/RTR-216), albeit the electron density was much weaker (Figs 3C and 4C). The average B-factor of NLS2-K residues 212–216 bound to P_1_ through P_5_ is slightly higher than importin α Arm-core (84 Å^2^ vs 50.0 Å^2^), but is nearly half than the B-factor at the minor NLS-binding site (120.0 Å^2^), where the electron density is very weak (Fig. 3C). Similarly, the refined B-factor of NLS-2R bound at the major NLS binding pocket is significantly lower than at the minor NLS pocket (76.0 Å^2^ vs 113.0 Å^2^). Thus, NLS2 is a genuine monopartite signal that targets the major NLS-binding site of importin α.

**Table 1:** Data collection and refinement statistics.

	ΔIBB-importin α1: NLS2-K (PDB ID: 5V5O)	ΔIBB-importin α1: NLS2-R (PDB ID: 5V5P)
Data collection		
Space group	P2_1_2_1_2_1_	P2_1_2_1_2_1_
Cell dimensions		
*a*, *b*, *c* (Å)	78.2, 90.9, 97.1	78.7, 91.3, 97.8
*α*, *β*, *γ* (°)	90.0, 90.0, 90.0	90.0, 90.0, 90.0
Resolution (Å)	50.0–2.25 (2.33–2.25)	50–2.10 (2.18–2.10)
*R*_sym_	8.6 (71.5)	8.4 (69.9)
*R*_pim_	4.6 (41.8)	4.1 (39.3)
*I*/σ*I*	29.5 (3.5)	32.8 (3.4)
Reflection (uni/tot)	32,858/127,285	42,795/641,609
Completeness (%)	97.8 (96.3)	99.3 (99.2)
Redundancy	3.9 (3.3)	5.0 (4.5)
Refinement		
Resolution (Å)	15–2.25	15–2.15
No. reflections	32,817	38,574
*R*_work_/*R*_free_	17.4/20.6	18.0/21.0
No. protein atoms	3,333	3,316
*B*-factors (Å^2^)		
Protein	51.8	47.9
NLS2 (P_1_- P_5_/P_1_′- P_5_′)	84.0/120.0	76.4/113.3
R.m.s. deviations		
Bond lengths (Å)	0.002	0.003
Bond angles (°)	0.53	0.53

Values in parentheses are for highest-resolution shell.

The R_free_ was calculated using 5% of randomly selected reflections.

### An arginine at position 214 weakness NLS2 interaction with importin α

To decipher why an R at position 214 results in decreased association with importin α and reduced nuclear import of 5GFP as compared to NLS-K (Fig. 2B,C), we carried structural alignment of NLS2 with other classical NLSs solved in complex with importin α (Table 2). This revealed the critical position P_2_ is occupied by R213 in both NP-NLS2-K and NP-NLS2-R, in stark contrast to all known NLSs that have a K at this position^8^. The lack of K at P_2_ in NLS2-K is likely compensated by K214, at position P_3_, which provides a register for correct positioning of the NLS at the major NLS-binding pocket. However, in NLS-2R both P_2_ and P_3_ are occupied by arginine residues (R213 at P_2_ and R214 at P_3_ position) (Table 2; Fig. 4B,C), possibly explaining why NLS2R is even weaker than NLS2-K. Likewise, the residues surrounding P_2_ and P_3_ are not likely to make a significant energetic contribution to the interaction with importin α. Position P_1_ lacks a side chain (G211), and T215 at position P_4_ coordinates a water molecule through the hydroxyl group. It is possible that phosphorylation of this residue enhances binding interaction with importin α, promoting nuclear import, as observed for certain phospho-NLSs^29^. Finally, position P_5_ is occupied by R216, which is sandwiched between W142 and W184 (Fig. 3C). Here the indole group of W142 makes a cation-π interaction^30^ with the guanidinium group of R216, while W184 stabilizes the aliphatic portion of R216. This interaction, which resembles that of the atypical NLS from phospholipid scramblase 1 (PLSCR1) that also binds only at the major NLS-binding site but nevertheless has a lysine at P_5_^31^, appears to be energetically very important. In summary, based on the lack of a lysine at position P_2_, we predict NLS2 of NP is a weak monopartite NLS.

**Table 2:** Structural alignment of importin α/Kap60 solved in complex with NLSs.

NLS type	Minor Binding Site P_-1_′P_0_′P_1_′P_2_′P_3_′P_4_′P_5_′		Major Binding Site P_1_ P_2_ P_3_ P_4_ P_5_	PDB id
SV40T-ag	P K K **K** R K		P K **K** K R K V	1EJL/1BK6*
hPLSCR1-NLS			G **K** I S K HWTGI	1Y2A
hPLSCR4-NLS	S I I **R** K W N			3Q5U
Guα-NLS	G Q K **R** S F S			3ZIN
A89-NLS	L G K **R** K Y W			4B8P**
B54-NLS	L G K **R** K R H			2YNS**
TPX2	K **R** K H	P V K M I K		3KND
C-Myc	K **R** V K L	P A A K R V K		1EE4*
Nucleoplasmin	A V K **R** P A A	TKKAG	Q A K **K** K K L D	1EJY/1EE5*
Kap60-IBB	E L R **R** R R D	TQQVELRKAKRDEA	L A **K** R R N F	1WA5*
h1NLS	TR K K **R** K D P	DSDDWSES	N S **K** E N K ID	4XZR*
h2NLS	T N K **R** K R E	QISTDNEAKMQIQEEKS	P K **K** K R K KRSSKANK	4PVZ*
NP-NLS1	SQGT K **R** S Y EQM			4ZDU
NP-NLS2-K	G R **K** T R		GE N G **R** K T R	5V5O
NP-NLS2-R	G R **R** T R		G **R** R T R	5V5P

*Denotes yeast importin α (Kap60). **Denotes rice importin α. In all other cases, mammalian importin α was co-crystallized with NLSs.

### Binding affinities of influenza NLSs for importin α1

**Figure 5:**
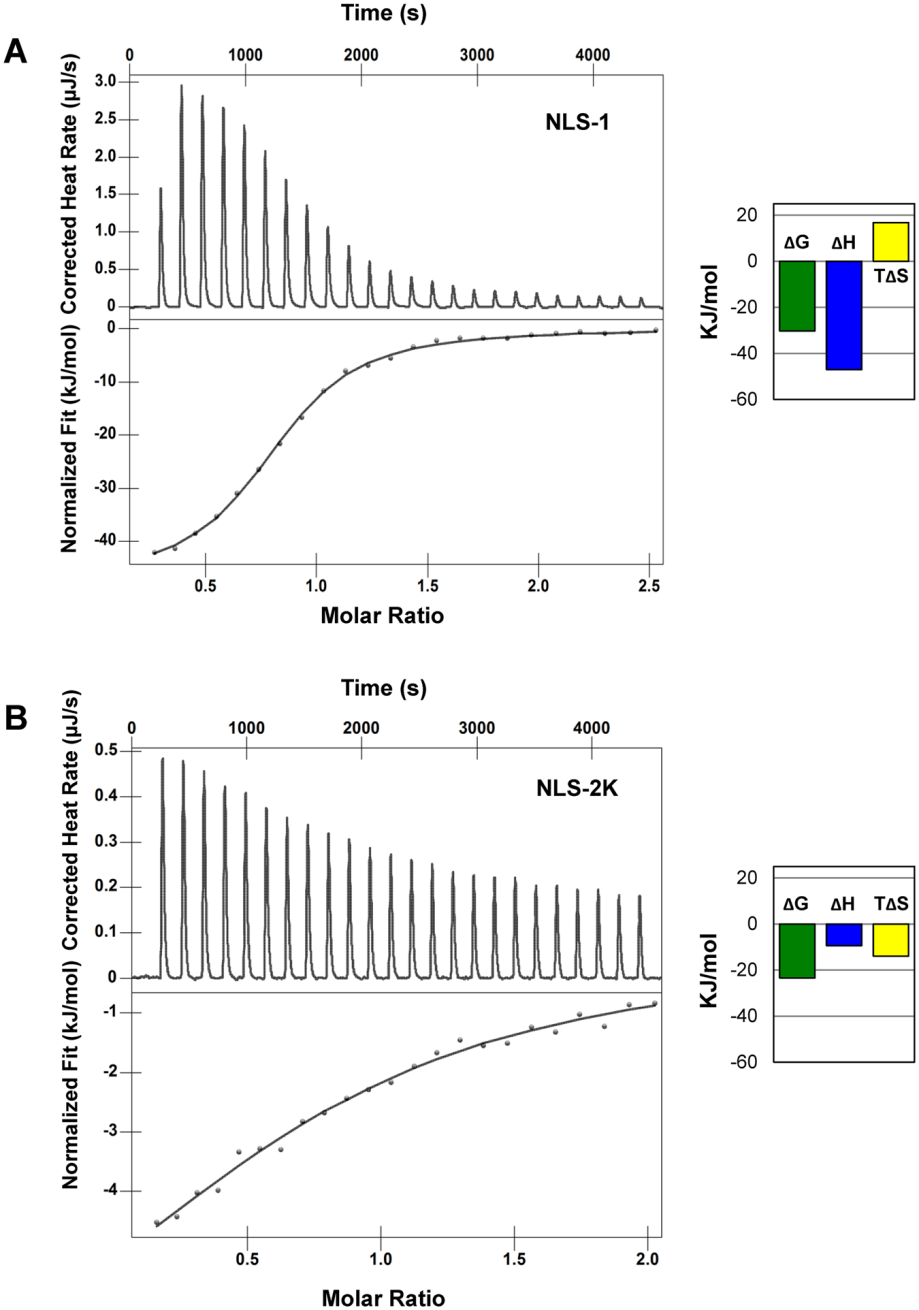
Calorimetric analysis of the interaction of influenza NLS1 and NLS2 with importin α1. ITC analysis of the interaction of ∆IBB-importin α (in the cuvette) with (**A**) NLS1 and (**B**) NLS2-K in the syringe. Raw data are in the top panels, and the integrated enthalpy plotted as a function of the NLS: ΔIBB-importin α molar ratio is shown in the bottom panels. The variation of enthalpy (∆H), entropy (T∆S), and Gibbs (T∆G) energy associated to each binding event are shown next to each panel.

The association of influenza NLS2 with importin α1 observed crystallographically (Figs 3 and 4) and previous reported data for NLS1^16^ prompted us to measure the binding affinities of influenza NLS1 and NLS2 for importin α in solution. Using nano Isothermal Titration Calorimetry (nano-ITC), we measured the heat released upon titration of increasing concentrations of NLS1 and NLS2 peptides inside a cuvette containing purified ∆IBB-importin α1. For NLS1, we observed a saturable endothermic reaction, which saturated within 15–16 injections after the NLS concentration in the cuvette was ~140 µM. Binding data were fit using a one binding site model yielding an equilibrium dissociation constant (K_d_) equal to 4.9 ± 0.4 µM (Fig. 5A), in good agreement with reported data^16^. At the same concentration of importin α in the cuvette and NLS in the syringe, NLS2-K gave a very small heat release, suggesting NLS2 is a weaker binder than NLS1. Increasing the concentration of NLS2-K peptide to ~215 µM, we obtained saturable data, which we fit to a one independent binding site model, with K_d_ = 72.4 ± 10.0 µM (Fig. 5B). Thus, NLS1 appears to be a stronger binder than NLS2. Using the two thermodynamic equations ∆G = ∆H − T∆S and ∆G = −RT ln(1/Kd), we also compared the thermodynamic parameters associated to NLS-binding to importin α. Interestingly, NLS1 association to importin α (Fig. 5A) involves negative values of ΔH and ΔS at the experimental temperature, indicating a balanced binding affinity based on both favorable hydrogen and van der Waals interactions and hydrophobic interactions. The entropic contribution likely reflects the involvement of three tryptophans (Trp) in importin α1 (Fig. 5A). In contrast, the binding affinity of NLS2-K for importin α (Fig. 5B) is based exclusively on hydrogen and van der Waals interactions (∆H < 0), but is accompanied by unfavorable entropy changes (∆S > 0), possibly due to conformational effects.

### NLS2 contributes to the binding of NP to different importin α isoforms

**Figure 6:**
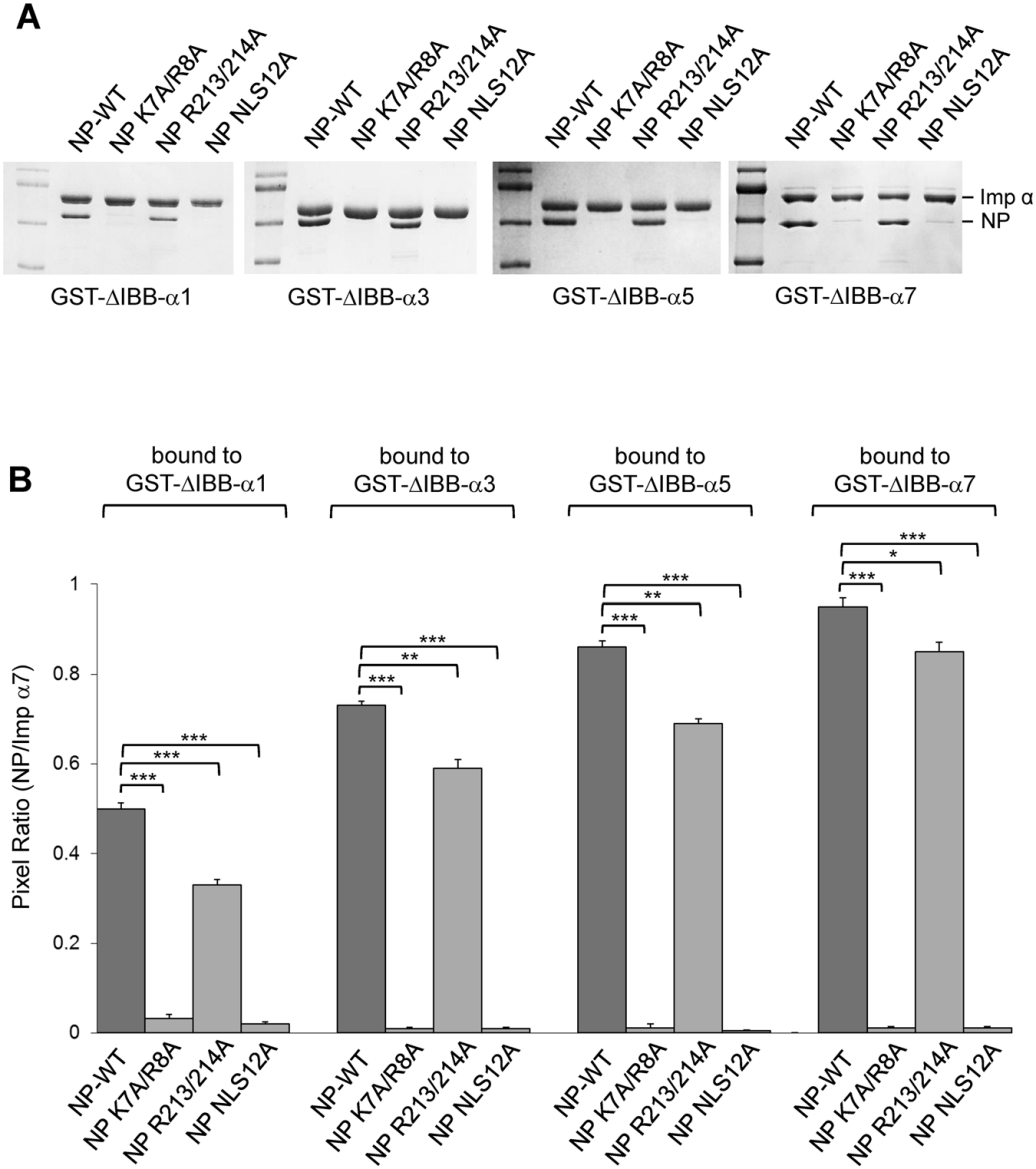
Pull-down analysis of the interaction of influenza A NP and importin α isoforms 1, 3, 5, and 7. (**A**) The gel shows equal volumes of GST-importin α:NP complex eluted from glutathione beads. (**B**) Quantification of pull-downs in panel A shown as mean ± SD for three independent experiments. One-way ANOVA followed by Tukey’s tests was used to determine significance, where *p < 0.05, **p < 0.01 and ***p < 0.001. Loading controls are shown in Fig. S4A. No interaction was observed between free NP/NP-mutants and GST (Fig. S4B).

The nucleoprotein of influenza A interacts with several isoforms of importin α, including the universal α1^12, 19, 20, 32^, and isoforms α3^12, 20, 32^ and α5^12, 20, 32^. Binding of NP to isoform α7 has not yet been tested, but since this isoform is required for efficient replication of influenza A virus in the respiratory tract^33^, we hypothesized NP also binds importin α7. We used a quantitative pull-down assay to determine how NLS2 contributes to the binding of NP to importin α isoforms and determine which isoform binds more efficiently to NP *in vitro*. Purified GST-tagged importin α1, α3, α5, and α7 (lacking the IBB) and NP were incubated at physiological concentration (~1 µM and 0.75 µM, respectively) and after reaching equilibrium, the complex was immobilized on glutathione beads, washed to remove unbound NP and eluted with reduced glutathione. Identical elution volumes were analyzed by SDS-PAGE and quantified in triplicates. Three NP mutants were also tested for binding to importin α isoforms: two NP mutants carrying Ala-mutations at P_2′_/P_3′_ and P_2_/P_3_ of NLS1 and NLS2, respectively, and one double mutant carrying mutations at both NLS1 (P_2′_/P_3′_) and NLS2 (P_2_/P_3_). Interestingly, we found that NP interacts with importins α1, α3, and α5, and to greatest extent with importin α7 (Fig. 6A). Nearly twice the quantity of importin α7 was recovered on gels as compared to importin α1 after incubation of equal concentrations of NP with either isoform (Fig. 6B). Point mutations in NLS1 greatly destabilized association of NP with all importin α isoforms, reducing the amount of recovered NP by 80 to 90%. Destruction of NLS2 alone also affected binding to NP, reducing binding to importin α1 and α7 by about 30% and 10%, respectively (Fig. 6B). In all cases, disruption of both NLSs completely abolished biding to importin α isoforms.

### Influenza NP is a RanGTP-dependent import cargo that overcomes IBB-autoinhibition

**Figure 7:**
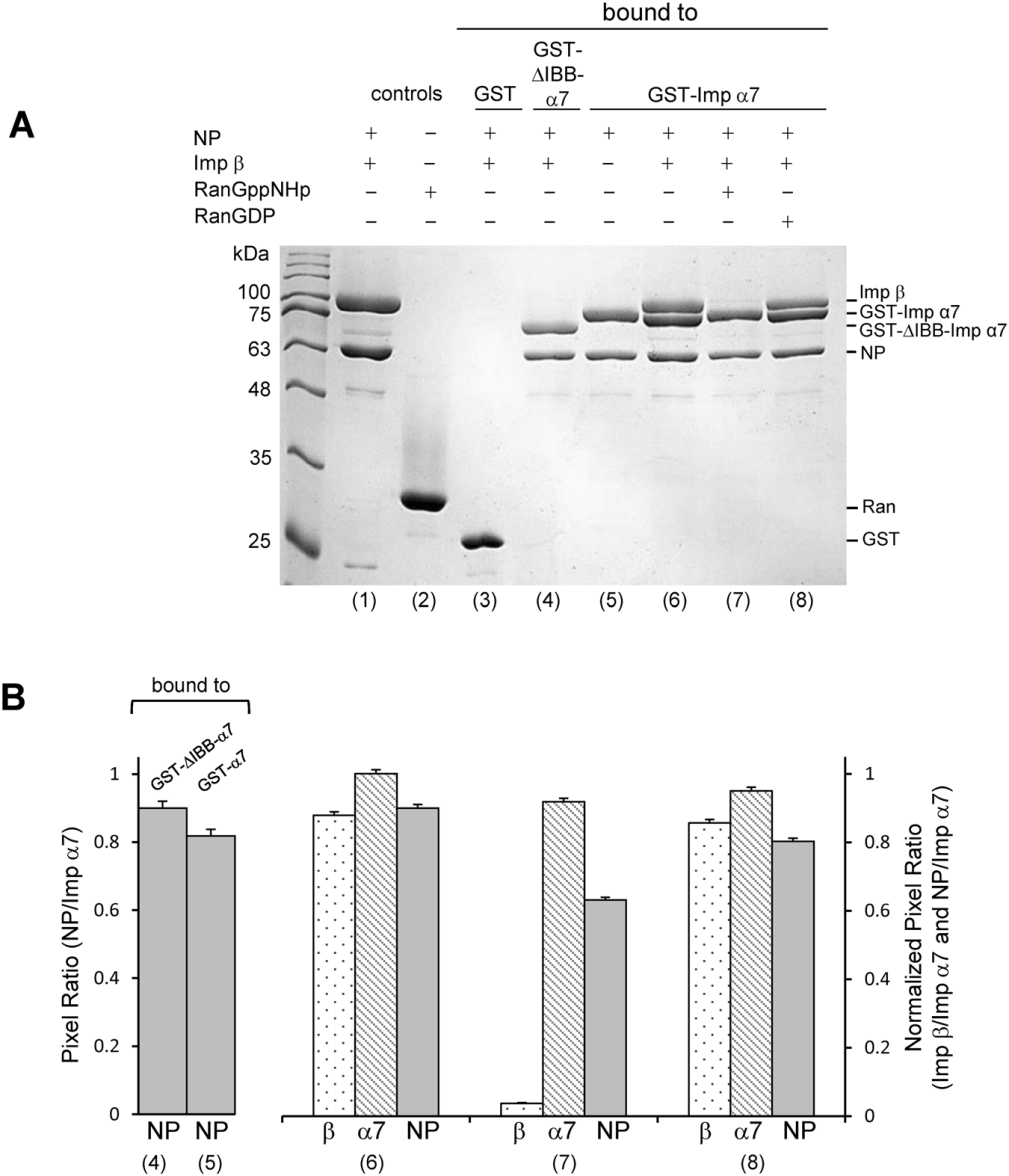
Influenza A NP nuclear import complex is disrupted by RanGppNHp. (**A**) Pull-down analysis of the interaction of NP with importin α7, importin β, and Ran. The gel shows equal elution volumes from glutathione beads coupled to free GST (lane 4), GST-ΔIBB-importin α7 (lane 5) or GST-importin α 7 (lane 6–8). (**B**) Quantification of pull-downs in panel A shown as mean ± SD for three independent experiments.

We assembled the NP import complex on glutathione beads by incubating purified NP with physiological concentrations^34^ of GST-importin α7 (1 μM) and importin β (3 μΜ) (Fig. 7A). We found that NP efficiently associates with the full length importin α7 in the absence of importin β suggesting its two NLSs can readily overcome IBB-autoinhibition (Fig. 7B). The pre-assembled GST-importin α7:importin β:NP importin complex was disrupted upon incubation with RanGppNHp but not RanGDP (Fig. 7B), suggesting NP is a RanGTP-dependent cargo. However, unlike classical NLS-cargos, NP remained bound to importin α7 after importin β displacement, confirming the IBB-domain of importin α7 is not sufficient to compete off NP NLSs from the Arm-core. This agrees with previous findings that importin α7 is only ~50% autoinhibited for classical NLS-cargos^35^.

## Discussion

Nuclear import of the influenza A viral genome is emerging as an important pharmacological target for the development of novel antiviral drugs that bears significant promise over classical vaccines and the current anti-influenza drugs^36^. To this end, a detail understanding of the nuclear import signals of NP and host importins implicated in the nuclear import of the influenza genome is of paramount importance.

Influenza NP has long been known to exploit the host import machinery. Over two decades ago, the Palese lab identified importin α as a host factor that co-purifies with influenza A NP, henceforth named ‘nucleoprotein interactor 1′ or NPI-1^19^. The strong interaction between NP and the host importin α is however incompatible with the weak micromolar binding affinity of NLS1 for importin α (Fig. 5A)^16^, suggesting additional binding determinants for importin α must exist in the structure of NP. In this paper, we have characterized the so-called second NLS of NP, or NLS2. Using a combination of functional, biochemical, and structural techniques, we made three fundamental discoveries.

*First*, NLS2 is functionally important in the context of a viral infection, and, *in vitro*, this import signal facilitates nuclear import of a non-diffusible chimeric protein. Through site-directed mutagenesis of the two clusters of basic amino acids and deletion of the C-terminal cluster, we found that NLS2 is not a bipartite NLS, as previously suggested^14^. Blocking NLS2 in infected cells significantly reduces production of progeny virion (Fig. 1B). Although this effect could be due to the role of NLS2 in viral replication^37^, the mislocalization of NP at 10 h p.i. (Fig. 1B,C) is a clear indication that NLS2 contributes to nuclear import during influenza A virus infection.

*Second*, NLS2 is a monopartite import signal that binds the major NLS-binding pocket of importin α, unlike NLS1 that is specific for the minor-NLS pocket^16^. We precisely mapped R213 of NLS2 as the residue occupying position P_2_ of the major NLS-binding pocket of importin α. P_2_, energetically the most critical residues of an NLS, is commonly occupied by K (Table 2)^8^, suggesting NLS2 is an intrinsically weak NLS. Also relatively uncommon in NLS2 is a strong contact between R216 at position P_5_ and W142 and W184 of importin α. NLS2-R, a second natural variant of NLS2 was even weaker that NLS2-K in our functional (Fig. 2B,C) and structural (Fig. 4) analysis. The reduced potency of this NLS is not readily explained by the presence of an R at position P_3_, which in mutational and peptide library studies was found to be energetically preferred over a K^8^. However, while the longer side chain of an R at P_3_ makes more favourable electrostatic interactions with Glu266/Asp270 of importin α, the lack of hydrogen-bonding interactions with these residues, may contribute to reduced binding specificity. The presence of three Rs in the span of four residues of NLS2-R may result in a register shift that puts R216 out of frame with importin α. This scenario is less likely in NLS-K, where a K at P_3_ provides a unique register to the Rs at P_2_ and P_5_.

*Third*, like NLS1, NLS2 is also a weak, micromolar binder of importin α. The combination of two import signals in NP results in high avidity for importin α, especially the isoforms α5 and α7, which belong to the importin α3-subfamily^38^. The equilibrium binding constants of NLS1 (K_d_ = 4.9 ± 0.4 µM) and NLS2 (K_d_ = 72.4 ± 10.0 µM) reported in this paper are significantly lower than those measured using fluorescence-depolarization^39^ or surface-immobilized NLSs^40^, but nonetheless consistent with published ITC studies that employed short NLS-peptides^29, 41–43^. Both Kds are significantly higher than the physiological concentration of importin α (~1 μM^34^), suggesting neither NLSs are sufficient to promote NP nuclear import.

**Figure 8:**
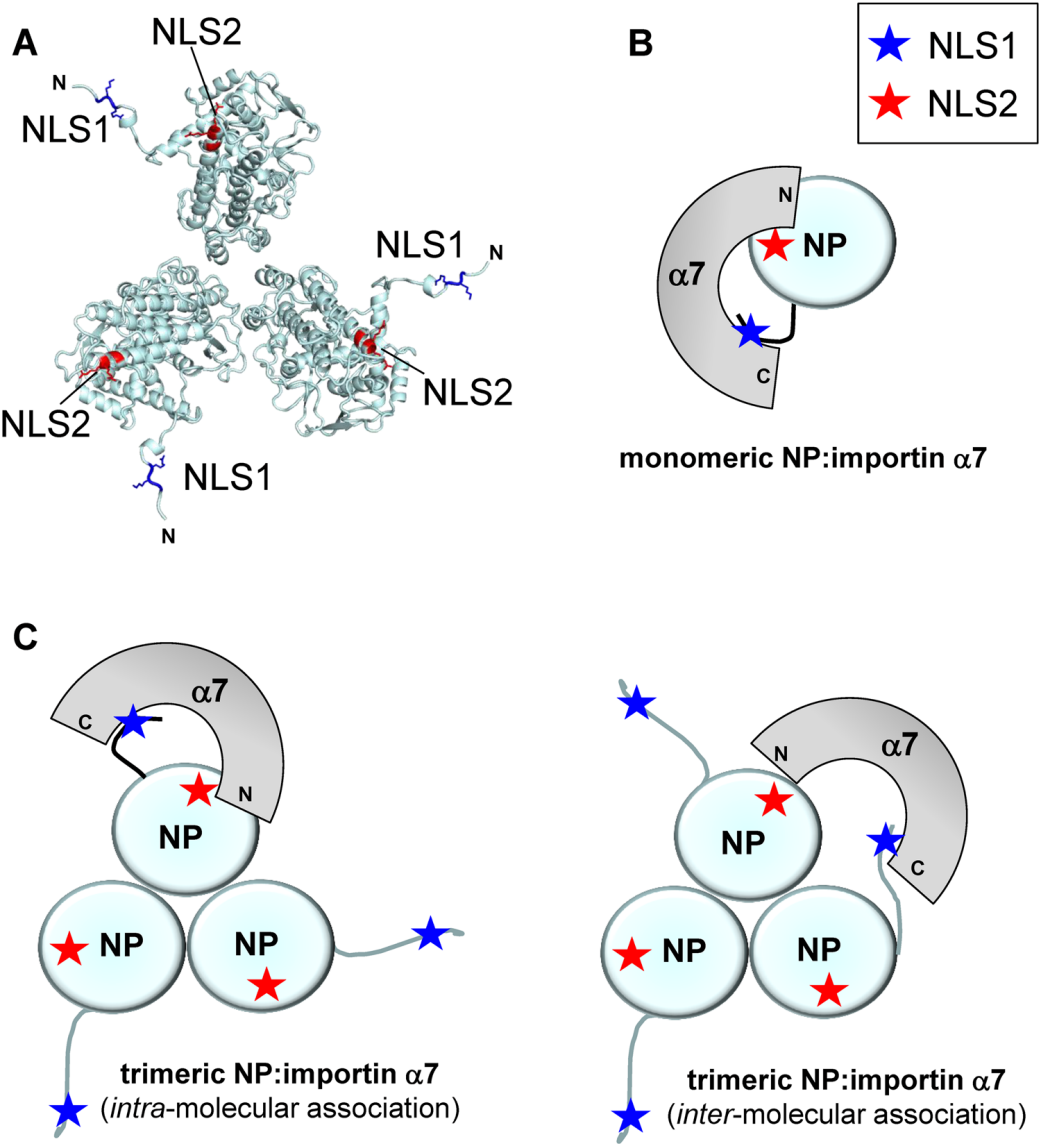
Model of NP bound to importin α7. (**A**) Structural model of the full length trimeric NP (colored in light cyan) based on pdb 2iqh. The position of NLS1 (residues 6–7) and NLS2 (residues 213–216) is indicated and residues at positions P_2_-P_3_/P_2_′-P_3_′ are colored in dark blue and red, respectively. (**B**) Schematic diagram of the monomeric NP (light cyan) bound to importin α7 (gray). NLS1 and NLS2 interacting with the minor and major NLS-binding pockets, respectively, are illustrated as stars color-coded as in panel A. (**C**) Schematic model of the trimeric NP (light cyan) bound to one equivalent of importin α7 (gray). Two possible modes of association as shown: *left* panel, importin α7 is bound to NLS1 and NLS2 from one NP protomer (*intra*-molecular association); *right* panel, importin α7 is bound to NLS1 and NLS2 from two NP protomers (*inter*-molecular association).

We propose NLS1 and NLS2 function *in trans* like a bipartite NLS that forms only in the tertiary (or quaternary) structure of NP (Fig. 8A) and interact simultaneously with the Arm-core of importin α occupying the minor and major NLS-binding pockets, respectively (Fig. 8B). The distance between the α-carbon positions of NLS1-K7 (at P_2_′) and NLS2-K214 (at P_2_) is about 30 Å, which is roughly equal to the distance between NLS1 and NLS2 in the 3D-structure of monomeric NP, although the N-terminal NLS1 was not visible in the crystal structure of NP^44^ and was computationally modelled. This model predicts that the overall avidity of NP for importin α is determined by the product of the Kds of each NLS for importin α^45^, explaining how two weak NLSs can function as a potent import signal if simultaneously bound to importin α. It is possible NP developed two weak NLSs, each bearing only 2–3 basic residues rather than 4–5 like most classical NLSs, in order to prevent non-specific association with cellular and viral RNA that could prevent association with importins and thus nuclear translocation. The conservation of multiple weak NLSs complementing *in trans* to increase the avidity for importin α was also proposed for the tripartite terminase complex of certain herpesviruses^43^ and may represent a recurring theme in virology.

NLS2 is conserved in all influenza A strains, reflecting an important and conserved function. NLS2 falls at the end of a α-helix in a region that can likely unfold upon binding the major NLS-binding site. It is not unusual for NLSs to exist in a helix-to-coiled equilibrium and switch to a coiled, stretched conformation upon binding to importin α. For instance, the IBB-domain of importin α is fully helical when bound to importin β^46^ but opens up like a bipartite NLS in its auto-inhibited conformation bound to the Arm-core^28^. Likewise, NF-κB p65 NLS, also within a helical region in complex with IκBα, functions like a classical importin α-dependent NLS^47^. Interestingly, removing NLS2 does not disrupt NLS1, but possibly slows down nuclear import, which is dominated by kinetics^48^. This is not readily appreciated *in vitro*, where disruption of NLS2 had little effect on nuclear accumulation of NP measured using a nuclear import assay in digitonin permeabilized cells^21^. However, *in vivo*, where the concentration of NP is typically low and NLS1 is outweighed by a plethora of much stronger cellular NLS-cargos, the synergy of two NLSs is likely essential for infection, especially in cells that have lower concentrations of importin α7, the isoform to which NP binds with the highest avidity (Fig. 6). In this respect, it should be noticed that NP mediates nuclear import at two different stages of infection. Early during infection, NLSs on NP facilitate the nuclear import of incoming vRNPs that must enter the nucleus for viral replication. NLSs on NP also mediate the nuclear import of newly synthesize NP, which is transported into the nucleus where is used to assemble progeny vRNPs. The oligomeric state of NP is different in these two stages of infection. NP forms a double helix packing of dimeric NPs in the vRNP that exposes NLS1^49^, and to some extent NLS2^23, 50^, while newly synthesized NP is trimeric in the absence of RNA^22^ and potentially exposes both NLSs (Fig. 8C). Thus, it is possible that two NLSs play different roles in the virus lifecycle, with NLS1 being more important for the nuclear import of vRNPs, due to the exposure of several copies on the vRNP structure, and NLS2 critical for the nuclear import of trimeric NP.

In conclusion, this paper expands our understanding of NP nuclear import by deciphering the structure and function of the NLS2. We demonstrate this weak NLS is active and functions in synergy with NLS1. This work solves a long-standing problem in the biology of influenza A virus nuclear transport and paves the way to decipher novel antivirals that disrupt nuclear import of NP.

## Methods

### Molecular biology techniques

The plasmid encoding five GFP molecules in tandem, generated by cloning in frame four GFP cDNAs into the pEGFP-C3 vector was a generous gift from Dr. Gergely L. Lukacs, McGill University^24, 51^. To generate the 5GFP-NLS1 and 5GFP-NLS2 constructs, the synthetic DNA for NLS1 and NLS2 from influenza A virus strain A/X-31 H3N2 containing adapters of the Bam HI restriction enzyme at each end (Table 3) were annealed, and the annealed DNA fragments were ligated to the Bam HI site at the C-terminal coding sequence of 5GFP. All constructs were confirmed by DNA sequencing. QuikChange site-directed mutagenesis kit (Stratagene) was used to generate mutations of the NLSs on 5GFP-NLS2; the 5GFP-NLS2 plasmid was used as the template to generate different mutants. For pull-down experiments substitution mutations in influenza A virus (H1N1 strain A/WSN/33) monomeric nucleoprotein at NLS1 and NLS 2 were generated by site directed mutagenesis. A total of three mutants were generated: (1) K7A-R8A to silent NLS1, (2) R213A-R214A to block NLS2, and (3) K7A-R8A and R213A-R214A to inactivate both the NLS1 and NLS2. All primers used in this study are listed in Table 3.

**Table 3:** Primers used in this study.

Construct name	Sequence (F indicates forward; R indicates reverse)
5GFP-NLS1	F: 5′-GATCCAATGGCGTCTCAAGGCACCAAACGATCATATGAACAATGCCG-3′
	R: 5′-GATCCGGCATTTGTTCATACGATCGTTTGGTGCCTTGAGACGCCATTG-3′
5GFP-NLS2	F: 5′-GATCCAAAACGTGGAATCAATGACCGAAATTTCTGGAGGGGTGAAAATGGACGAAAGACAAGGG-3′
	R: 5′-GATCCCCTTGTCTTTCGTCCATTTTCACCCCTCCAGAAATTTCGGTCATTGATTCCACGTTTTG-3′
5GFP-NLS2 A1	F: 5′-CTGTACAAGCAGGATCCAGCAGCTGGAATCAATGACCG-3′
	R: 5′-CGGTCATTGATTCCAGCTGCTGGATCCTGCTTGTACAG-3′
5GFP-NLS2 A2	F: 5′-GAGGGGTGAAAATGGAGCAAAGACAGCGCCGAATC-3′
	R: 5′-GATCCGGCGCTGTCTTTGCTCCATTTTCACCCCTC-3′
5GFP-NLS2 DC	F: 5′-GATCCAAAACGTGGAATCAATGACCGAAATTTCTGGG-3′
	R: 5′-GATCCCCAGAAATTTCGGTCATTGATTCCACGTTTTG-3′
NLS1-K7A-R8A	F: 5′-GCGACCAAAGGCACCGCAGCATCTTACGAACAGATG-3′
	R: 5′-CATCTGTTCGTAAGATGCTGCGGTGCCTTTGGTCGC-3′
NLS2-R213A-R214A	F: 5′-AGGGGTGAGAATGGAGCAGCAACAAGGATTGCTTAT-3′
	R: 5′-ATAAGCAATCCTTGTTGCTGCTCCATTCTCACCCCT-3′

### Biochemical techniques

Importin α isoforms α1, α3, α5 and α7 lacking the N-terminal IBB-domain were expressed in bacteria and purified as previously described^35, 41, 52^. All importin α isoforms were purified over a Superdex 200 column (GE Healthcare) equilibrated in Gel Filtration (G.F.) buffer (20 mM Tris pH 8.0, 150 mM NaCl, 5 mM β-mercaptoethanol, and 0.2 mM PMSF). Purified ΔIBB-importin α isoforms were concentrated to 20 mg/ml using a Millipore concentrator (cut-off 10 kDa). Expression and purification of human importin β and Ran loaded with GDP (RanGDP) or nonhydrolyzable GTP (RanGppNHp) were carried out as described^53^. Peptides encompassing influenza NLS1 (1-MASQGTKRSYEQM-13), NLS2-K (198-KRGINDRNFWRGENGR**K**TR-216) and NLS2-R (198-KRGINDRNFWRGENGR**R**TR-216) were custom synthesized (GenScript) and purified at 95% homogeneity by reverse phase chromatography. Influenza A virus nucleoprotein and mutants were expressed in *Escherichia coli* BL21 (DE3) cells and purified using a previously reported procedure^44^.

### Cell culture, transfection

HeLa cells were maintained at 37 °C and 5% CO_2_ in Dulbecco’s modified Eagel’s medium (DMEM) supplemented with 10% fetal bovine serum (FBS), 1% penicillin/streptomycin, 1% L-glutamine, and 1% sodium pyruvate. HeLa cells grown as monolayers on glass microscope coverslips were transfected with 5GFP or 5GFP-NLSs plasmids using Lipofectamine 2000 (Invitrogen) according to the manufacturer’s instruction.

### Imaging of transfected cells

Twenty-four h after transfection the cells were fixed with 3% paraformaldehyde in phosphate buffered saline (PBS) for 15 minutes at room temperature. Cells were then washed with PBS three times, fixed with 3% paraformaldehyde (PFA), and the coverslips were mounted onto microscope slides. Samples were visualized using a Fluoview FV1000 confocal laser-scanning microscope (Olympus).

### Infection and imaging of infected cells

HeLa cells were seeded on glass microscope coverslips and transfected with 5GFP or 5GFP-NLSs using Lipofectamine 2000 (Invitrogen) according to the manufacturer’s instruction. Twenty-four h post transfection, cells were infected with purified influenza A virus (X-31, A/Aichi/68 (H3N2); Charles River Laboratories) at a multiplicity of infection (MOI) of 4 in DMEM supplemented with 0.2% FBS. Cells were incubated for 15 minutes at 4 °C to allow the virus to bind to the cell surface. Cells were then moved to 37 °C for 1 h to allow virus internalization. After this incubation period, a mild acidic wash (PBS-HCl, pH 5.5 at 4 °C) was performed to exclude the delayed uptake of attached, but not internalized virus particles. Subsequently, cells were incubated at 37 °C in DMEM supplemented with 2% FBS for 1 h and 9 h, respectively. Cells were then washed with PBS three times, fixed with 3% PFA for 15 minutes, permeabilized with 0.2% Triton X-100 in PBS for 5 minutes, and incubated with PBS containing 1% bovine serum albumin (BSA) and 10% goat serum for 1 h at room temperature. Cells were then incubated with mouse NP monoclonal antibody (1:1000 dilution) (Novus Biologicals, Cat. #NB10–56570) for 1 h at 37 °C, washed with PBS containing 2.5% BSA, and incubated with goat anti-mouse conjugated with Alexa Fluor 568 (1:2000 dilution) for 45 minutes at room temperature. Coverslips were mounted in Prolong Gold antifade reagent containing DAPI. Samples were visualized using an Olympus Fluoview FV1000 laser-scanning microscope.

### Plaque assay

Supernatant were obtained from infected cells after 24 h infection and used to evaluate virus titers. Madin-Darby canine kidney (MDCK) cells were seeded in 6-well plates at a density of 7 × 10^5^ cells/well 48 h before plaque assay. Supernatants containing virus progeny were serially diluted using 10-fold dilutions. Two hundred µl of each dilution was added to each well in triplicates. The plates were incubated at room temperature for 1 h on a shaker. After two times wash with PBS, 2 ml of nutrient agar overlay (1% agarose, 0.5% penicillin in Minimum Essential Medium Eagle) was added to each well and the cells were incubated at 37 °C in 5% CO_2_ for 72 h. Next, cells were fixed with 4% PFA and stained with 1% crystal violet in 20% methanol. The plaques were counted and used for viral titer calculation. The virus titers were expressed as plaque forming units (PFU)/ml = [(numbers of plaques per well) X dilution]/(inoculum volume).

### Quantification of nuclear import

To quantify the nuclear import of chimeric proteins, the ratio of the nuclear to cytoplasmic fluorescence signal was determined as previously described^23, 50^. Briefly, the mean intensity of a defined area in the nucleus was measured and divided by the mean intensity of the same size area in the cytoplasm from the same cell using ImageJ (National Institute of Health). The fluorescence of the nuclear envelope was not included in the quantification. After correction for background fluorescence, the results were expressed as the ratio of nuclear to cytoplasmic fluorescence (Fn/c). Data was obtained from a total of 85–100 cells per experiment from three independent experiments. Results were analyzed by One-way ANOVA followed by Tukey’s test using GraphPad Prism (GraphPad Software, Inc., La Jolla, CA). All data are represented as the mean value ± standard error of the mean and p < 0.05 were considered significant.

### Crystallographic Methods

Crystals of ∆IBB-importin α1 bound to NLS2 were obtained by mixing 2.3 µl of gel filtration-purified importin α1 at 18.0 mg ml^−1^ and 0.7 µl (3-fold molar excess) of peptide with an equal volume of 0.1 M HEPES buffer (pH 6.0), 0.6 M sodium citrate tribasic ehydrate, 10 mM β-mercaptoethanol and equilibrated against 600 µl of the same precipitant, at 18 °C. Crystals were harvested in nylon cryo-loops, cryo-protected with 27% ethylene glycol and plunged in liquid nitrogen. Crystals were diffracted at LS-CAT Beamline 21-ID-F at Argonne Photon Source on a MARMOSAIC 225 CCD detector and at beamline 14–1 at SSRL on a Rayonix MX325 CCD detector. Data indexing, integration and scaling were carried out with the HKL2000^54^. Initial phases were obtained by molecular replacement using Phaser^55^ and PDB entry 3Q5U as a search model. Atomic models were built using Coot^56^ and refined using *phenix.refine* ^57^. Data collection and refinement statistics are summarized in Table 1. All structural illustrations were carried out using PyMOL^58^.

### Isothermal Titration Calorimetry

ITC experiments were carried out at 25 °C using a nano-ITC calorimeter (TA Instruments)^43^. Influenza NLS1 and NLS2-K peptides were dissolved in G.F. buffer between 600–850 μM and injected in 2 μl increments into a calorimetric cell containing 195 µl of ΔIBB-importin α1 at 120 μM. The spacing between injections was 300 seconds. Titration data were analyzed using the NanoAnalyze data analysis software (TA Instruments). Heats of dilution were determined from control experiments with the ITC buffer and subtracted prior to curve fitting using a single set of binding sites model.

### GST-pull down assay

All pull-downs were carried out using physiological concentrations^34^ of purified GST-importin α isoforms (1 μM) (with or without IBB), importin β (3 μM), RanGppNHp or RanGDP (5 μM) and influenza NP (0.75 µM). Pull-downs with different isoforms in Fig. 6A were carried out by pre-incubated GST-importin α isoforms with NP, or relative mutants, for 30 minutes at 4 °C. 700 µl of this mixture was then incubated with 100 µl of glutathione resin beads (Pierce GST spin purification kit) for 1 hour at 4 °C. Beads were then washed 3 times with 700 µl of G.F. buffer and GST-importin α:NP complexes were eluted using 100 µl of G.F. buffer containing 25 mM reduced glutathione. For pull-downs in Fig. 7A, the NP import complex was formed in solution by incubating GST-importin α7, importin β and NP for 30 minutes at 4 °C. The complex was then captured on glutathione beads, which were washed three times with 700 µl of G.F. buffer. 700 µl of RanGppNHp or RanGDP (at 5 μM final concentration) were added to the beads and incubated for 1 hour at 4 °C followed by 3 washes with 700 µl of G.F. buffer and elution with 100 µl of G.F. buffer containing 25 mM reduced glutathione. All elution samples were analyzed by SDS-PAGE (15%), stained with Coomassie Brilliant Blue-G-250, destained overnight and quantified. ImageJ software^59^ was used to quantify all the bands and data are presented as pixel ratios. Briefly, the pixel density of each NP/mutant band in Fig. 6A was divided by the pixel density of the respective importin α isoform to obtain a pixel ratio. Similarly for quantification of Fig. 7A, the pixel density of importin α7 was divided by the pixel density of importin β (in the presence of RanGppNHp or RanGDP). Finally, error bars are presented as mean ± SD from three independent experiments. Results were analyzed by one-way ANOVA followed by Tukey’s test using Origin software^60^.

## Electronic supplementary material

Supplementary information
