# Supplementary material for: Synergy of two low-affinity NLSs determines the high avidity of influenza A virus nucleoprotein NP for human importin α isoforms
Source: Sci Rep. 2017 Sep 12;7:11381. doi: 10.1038/s41598-017-11018-1 (PMC5595889; doi:10.1038/s41598-017-11018-1)
Supplement: Supplementary file 1 — Supplementary information [file 41598_2017_11018_MOESM1_ESM.pdf]

Synergy of two low-affinity NLSs determines the high avidity of influenza A virus nucleoprotein NP for human importin α isoforms

Wei Wu[1,+], Rajeshwer S. Sankhala[2,+], Tyler J. Florio[2], Lixin Zhou[1], Nhan L.T. Nguyen[1], Ravi K. Lokareddy[2], Gino Cingolani[2,3*], and Nelly Panté[1,*]

[1]University of British Columbia, Department of Zoology, Vancouver, British Columbia, V6T1Z4, Canada

[2]Thomas Jefferson University, Department of Biochemistry and Molecular Biology, Philadelphia, PA 19107, USA

[3]Institute of Biomembranes and Bioenergetics, National Research Council, Via Amendola 165/A, 70126 Bari, Italy

[*] corresponding authors: gino.cingolani@jefferson.edu; pante@zoology.ubc.ca

[+] these authors contributed equally to this work

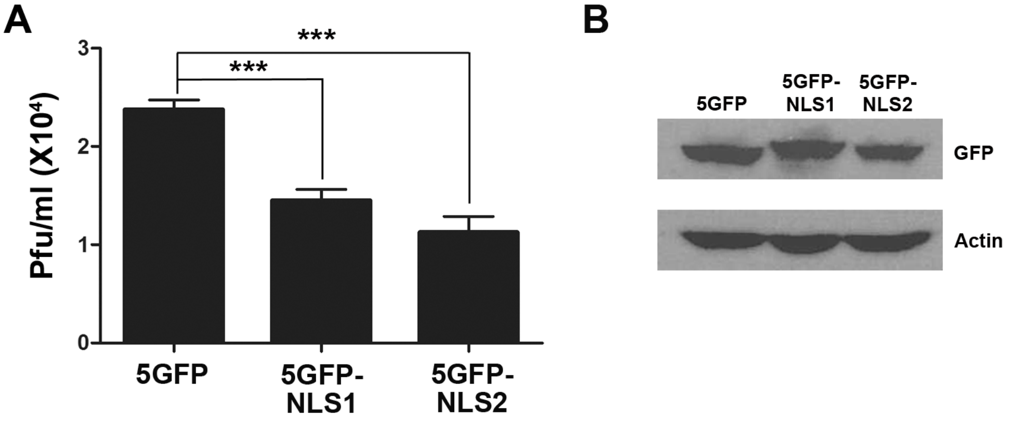

Figure S1: Infection with influenza A virus strain PR8 is reduced in cells expressing NLS1 or NLS2. (**A**) HeLa cells expressing 5GFP, 5GFP-NLS1, or 5GFP-NLS2 were infected with influenza A virus (strain A/PR8/1934/H1N1). At 24 h post-infection, the supernatant was collected and the viral titer was determined by infecting MDCK cells and counting plaques 3 days post-infection. Bar graphs show the mean ± standard error of the mean from three independent experiments (***$p<0.001$, one-way ANOVA followed by Tukey's tests). (**B**) Western blots of whole cell lysates from cells transfected with 5GFP, 5GFP-NLS1, or 5GFP-NLS2 for 24 h and infected with influenza A virus (strain A/PR8/1934/H1N1) for 24 h. Antibodies used were anti-GFP (Invitrogen, A11121, 1:10000 dilution) and anti-beta actin (Sigma, A4700, 1:5000 dilution).

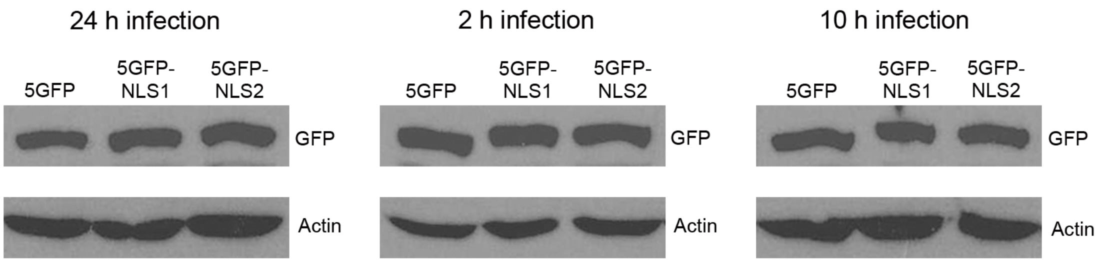

Figure S2: Levels of expression of 5GFP, 5GFP-NLS1, and 5GFP-NLS2 for experiments showed in Figs. 1B and 1C. Western blots of whole cell lysates from cells transfected with 5GFP, 5GFP-NLS1, or 5GFP-NLS2 for 24 h and infected with influenza A virus (strain X-31, A/Aichi/68 (H3N2)) for 24 h, 2 h, or 10 h. Antibodies used were anti-GFP (Invitrogen, A11121, 1:10000 dilution) and anti-beta actin (Sigma, A4700, 1:5000 dilution).

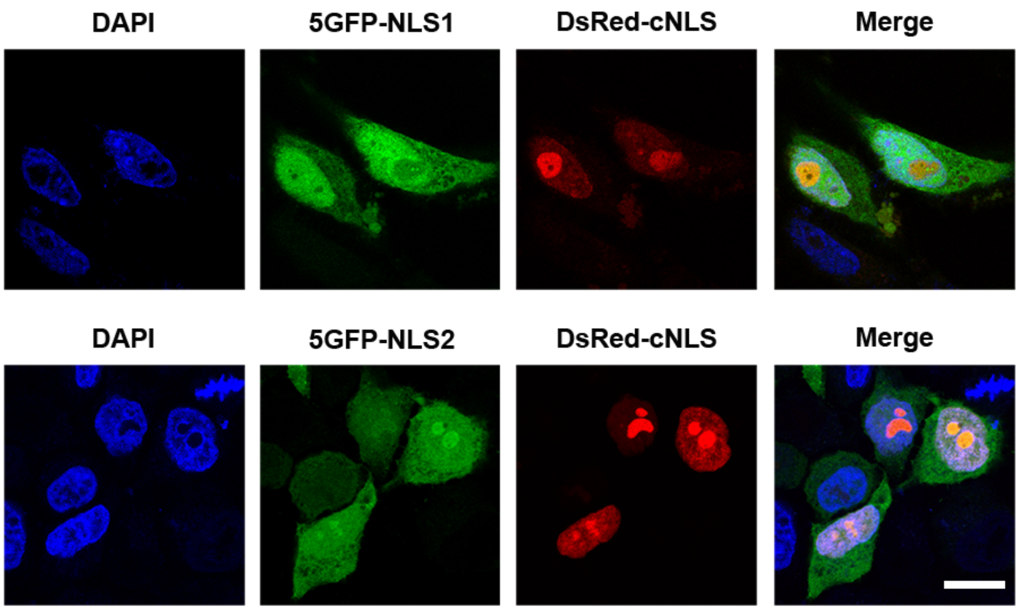

Figure S3: 5GFP-NLS1 and 5GFP-NLS2 do not affect the nuclear import of DsRed-cNLS. HeLa cells were co-transfected with plasmids expressing DsRed-cNLS (DsRed fused to three copies of the classical NLS of SV40 T antigen; Clontech Laboratories) and 5GFP-NLS1 or 5GFP-NLS2. The cells were visualized by confocal microscopy 24 h post transfection. DNA was detected by staining with DAPI. Scale bar, 10 μm. DAPI, blue; 5GFP-NLSs, green; DsRed-cNLS, red.

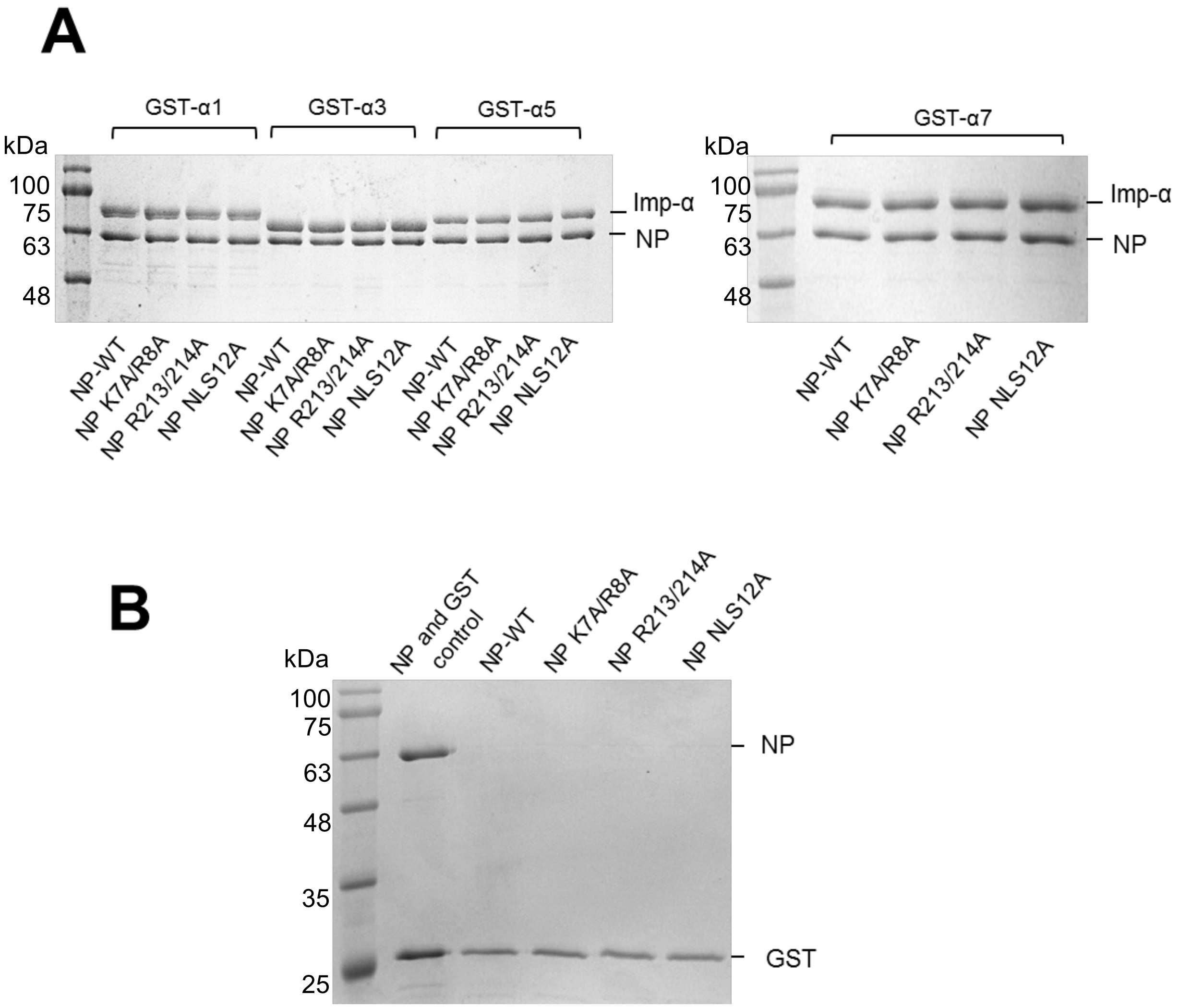

Figure S4: Control pull-downs. (**A**) 1 μM GST-tagged importin α isoforms 1, 3, 5, and 7 were used to pull down 0.75 μM His-tag NP and mutants. (**B**) 1 μM free GST was used to pull down 0.75 μM His-tagged NP and mutants.
